# Persistently elevated AD risk in hiPSC‐derived cortical neurons after developmental and adult Pb exposure

**DOI:** 10.1002/alz.090098

**Published:** 2025-01-03

**Authors:** Junkai Xie, Han Zhao, Shichen Wu, Aaron B Bowman, Jeniffer Freeman, Chongli Yuan

**Affiliations:** ^1^ Purdue University, West Lafayette, IN USA

## Abstract

**Background:**

Exposure to environmental chemicals such as lead (Pb) during vulnerable developmental periods and even in adult stage can result in adverse health outcomes later in life. Human cohort studies have demonstrated associations between Pb exposure and Alzheimer’s Disease (AD) onset in later life which were further corroborated by findings from animal studies. The molecular pathway linking Pb exposure and increased AD risk, however, remains elusive.

**Method:**

In this work, we used human iPSC‐derived cortical neurons as a model system to study the effects of Pb exposure on AD‐like pathogenesis in human cortical neurons. We exposed neural progenitor cells and differentiated neurons derived from human iPSC to Pb concentrations of 0, 15, and 50 ppb for 48 hours, simulating developmental and adult Pb exposure, respectively. Various techniques, including immunofluorescence, Western blotting, RNA‐sequencing, enzyme‐linked immunosorbent assay (ELISA), microelectrode array (MEA), and Förster resonance energy transfer (FRET) reporter cell lines, were employed to assess changes in AD‐like pathogenesis in differentiated cortical neurons. The susceptibility of Pb‐exposed neurons to cellular stressors such as PHF‐Tau and MPP+ was evaluated through secondary stress assays.

**Result:**

Exposing neural progenitor cells to low dose Pb, mimicking a developmental exposure can result in altered neurite morphology. Differentiated neurons exhibit altered calcium homeostasis, synaptic plasticity, epigenetic landscape along with elevated AD‐like pathogenesis markers, including phosphorylated Tau, Tau aggregates and Aβ42/40. Furthermore, Pb‐exposed cortical neurons exhibited significantly increased calcium dynamics and overall neuronal activity. Adult neurons exposed to Pb demonstrated heightened vulnerability to PHF‐Tau and MPP+‐induced cytotoxicity, with these changes persisting even after Pb withdrawal.

**Conclusion:**

Collectively, our findings propose a plausible molecular mechanism to account for the increased risk of AD in populations with a history of Pb exposure.

